# Photoexcited Enzymes for Asymmetric C*sp* ^3^−C*sp* ^3^ Cross‐Electrophile Couplings

**DOI:** 10.1002/anie.202214313

**Published:** 2022-11-10

**Authors:** Sandy Schmidt

**Affiliations:** ^1^ Department of Chemical and Pharmaceutical Biology Groningen Research Institute of Pharmacy Antonius Deusinglaan 1 9713 AV Groningen (The Netherlands

**Keywords:** Abiological Transformations, Biocatalysis, Cross Couplings, Photocatalysis, ‘Ene’-Reductases

## Abstract

Enzymes have several advantages over conventional catalysts for organic synthesis. Over the last two decades, much effort has been made to further extend the scope of biocatalytic reactions available to synthetic chemists, particularly by expanding the repertoire of enzymes for abiological transformations. In this regard, exciting new developments in the area of photobiocatalysis enable now the introduction of non‐natural reactivity in enzymes to solve long‐standing synthetic challenges. A recently described example from the Hyster group demonstrates in an unprecedented way how the combination of photochemistry with enzyme catalysis empowers the catalytic asymmetric construction of C*sp* ^3^−C*sp* ^3^ bonds with high chemo‐ and enantioselectivity.

## Graphical abstract

The combination of photochemistry with enzyme catalysis offers exciting opportunities to induce new reactivities and to create novel enzymes for reactions other than their native ones. Recently, Hyster and co‐workers demonstrated this for a photoenzymatic asymmetric C*sp* ^3^−C*sp* ^3^ cross‐electrophile coupling, a reactivity previously unknown to enzymes.

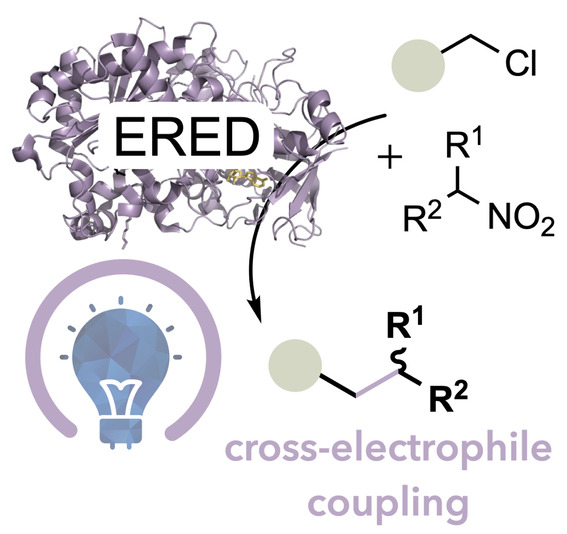

## Accessing Abiological Enzymatic Transformations

Enzyme catalysis intrinsically offers several advantages over traditional synthesis routes. Thus, biocatalysts are increasingly adopted by the pharmaceutical and chemical industries. However, many synthetically useful reactions cannot be performed with naturally occurring enzymes, limiting their expanded use in chemical synthesis. In the quest to expand the repertoire of enzymes for abiological transformations, different concepts have been devised and emerged into a hotbed of research.

**Figure 1:**
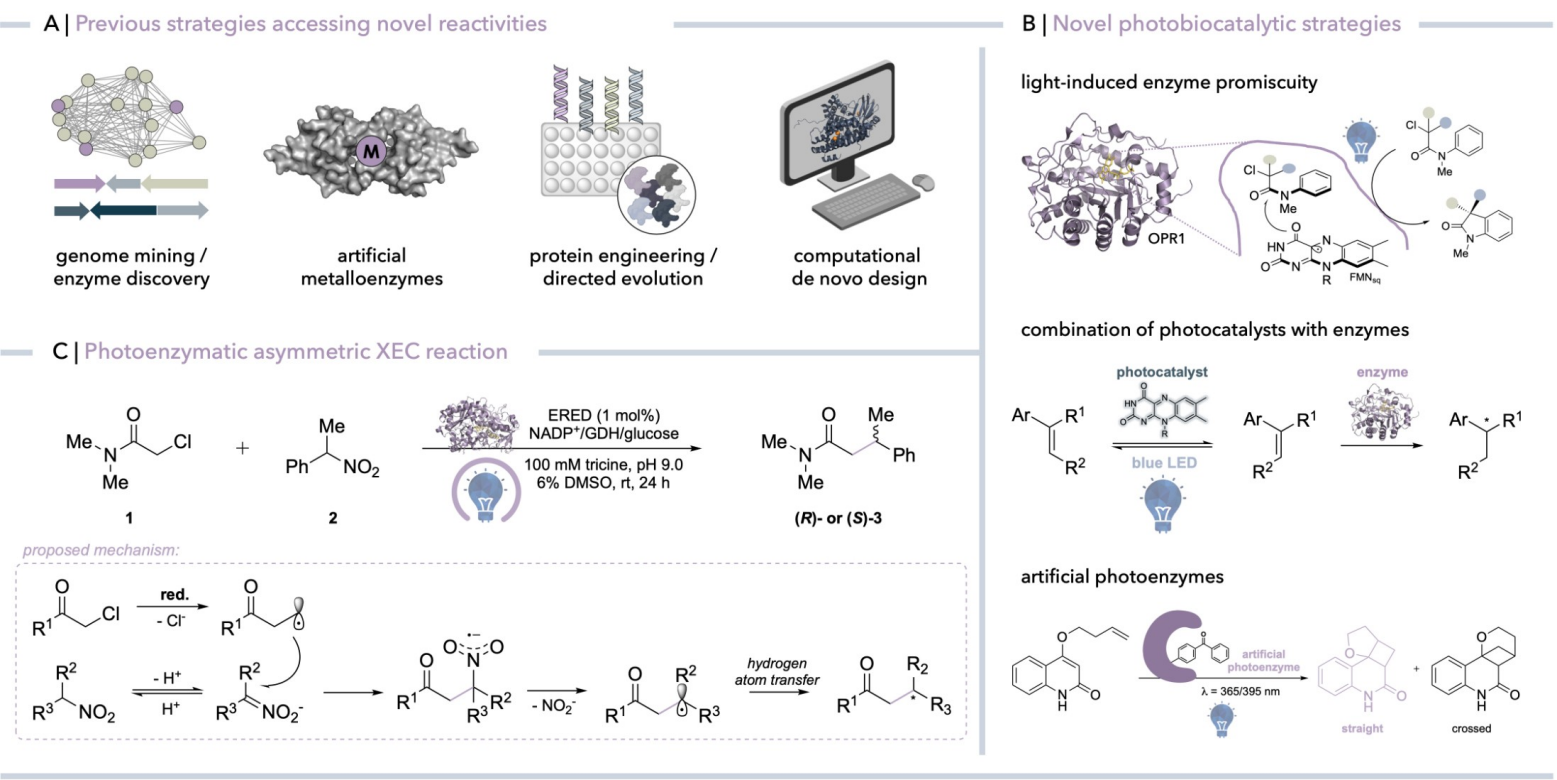
a) Several strategies have been devised in the past to develop enzymes that catalyze novel reaction types. b) Emerging concepts comprising the combination of photochemistry and enzyme catalysis to induce abiological reaction chemistries in enzymes.[^10^, ^11^, ^12^, ^13^] c) Photoenzymatic asymmetric cross‐electrophile coupling (XEC) developed by Hyster and co‐workers.^[15]^ Ar, aryl; OPR1, 12‐oxophytodienoate reductase; ERED, ‘ene’‐reductase; GDH, glucose dehydrogenase; NADP^+^, nicotinamide adenine dinucleotide phosphate; red., reduction.

One of those concepts follows a rather classical approach by mining nature to discover new types of chemical reactivity (Figure 1a).^[1]^ While in principle a powerful approach, it is intrinsically limited to reactivity patterns that nature invented. Thus, researchers now go one step further by developing strategies in which these reaction patterns are expanded to those currently unknown to nature.^[2]^ An early example that has facilitated an abiological reaction in a known protein scaffold was reported in 1978.^[3]^ The incorporation of a rhodium catalyst in avidin resulted in an asymmetric hydrogenation catalyst with modest enantioselectivity. Ever since many artificial metalloenzymes (ArMs) have been created for various transformations involving different metals, reaction mechanisms, and reactants (Figure 1a).^[4]^ Another strategy is to use protein engineering to redesign enzymes for reactions other than their native ones. Particularly, metal‐dependent enzymes such as P450 monooxygenases (P450s) or non‐heme iron‐dependent oxygenases have proven themselves as powerful scaffolds to access novel reaction chemistries (Figure 1a).^[5]^ The successfulness of this strategy is exemplified by the engineering of a heme‐dependent cytochrome c to catalyze carbon‐silicon bond formations^[6]^ or by tailoring P450 BM3 to catalyze stereoselective cyclopropanations^[7]^ or aziridinations.^[8]^ In addition to starting from an existing protein scaffold to access abiological reactions via protein engineering, computational *de novo* design has been used to create an enzyme that catalyzes a Kemp elimination reaction (Figure 1a).^[9]^

In the past few years, a striking new concept has been developed to introduce non‐natural reactivity in enzymes: combining biocatalysis and photochemistry allowing for selective abiological transformations fueled by visible light (Figure 1b).^[10]^ Although this is a relatively new area of research, many exciting examples already show how non‐natural reaction chemistries can be tapped either by light‐induced enzyme promiscuity, the design of artificial photoenzymes, or the direct combination of photocatalysts with enzymes in so‐called photochemoenzymatic reactions.[^11^, ^12^, ^13^] For instance, a recent example reported by the Green group highlights how genetic code expansion is used to develop a photoenzyme catalyzing enantioselective [2+2]‐cycloadditions via triplet energy transfer catalysis, thereby accessing a mode of reactivity that is new to biocatalysis (Figure 1b).^[13]^

## Illuminating ‘Ene’‐Reductases for New Reactivities

Previously, Hyster and co‐workers developed strategies via photoexcitation to form carbon‐centered radicals from organohalides, using flavin‐dependent ‘ene’‐reductases (EREDs),^[14]^ demonstrating the ability of this enzyme family to control the stereochemical outcome of radical reactions.

Now, the Hyster group further expands this approach to an ERED‐based photoenzymatic platform capable of harnessing an enzyme‐templated charge‐transfer (CT) complex for chemo‐ and enantioselective C*sp* ^3^−C*sp* ^3^ cross‐electrophile couplings (XECs) between alkyl halides and nitroalkanes (Figure 1c).^[15]^ The proposed reaction mechanism, comprising photoinduced radical initiation, C−C bond formation, C−N bond mesolytic cleavage and hydrogen‐atom transfer (HAT), is highly attractive, as the orthogonal reactivity of nitroalkanes and alkyl halides avoids undesired dimerization products and thus proceeds with high control of chemoselectivity. While the proposed initial reduction of the alkyl halide forms an alkyl radical that reacts with the in situ formed nitronate to forge a new C−C bond and a nitro radical anion, the enzyme mediates homolytic cleavage of the C−N bond generating nitrite and an alkyl radical that can be terminated by means of HAT, affording the cross‐coupled product (Figure 1c).

In this reaction, the ERED fulfills two particular functions, making this approach so powerful. First, by using light to excite the enzyme‐templated CT complex formed between the alkyl halides and the flavin cofactor, the chemoselective reduction of the alkyl halide over the thermodynamically favored nitroalkane partner is enabled. Secondly, the ERED is precisely controlling the radical‐terminating HAT step and is thus forming the cross‐coupled product with high enantioselectivity.

Astonishingly, many of the initially screened EREDs for the selected model reaction between α‐chloroamide **1** and 1‐nitroethylbenzene **2** afforded the desired cross‐coupled product. By choosing two stereocomplementary EREDs, both enantiomers of product **3** were accessible. Interestingly, performing the reaction with the photocatalyst Ir(ppy)_3_ instead of the ERED, the nitroalkane reduction to the oxime was observed, highlighting that this reactivity is unique to biocatalysis. After having optimized the reaction conditions, the authors sought to explore the scope and limitations of this photoenzymatic transformation. For instance, a variety of nitroalkanes were screened for their potential to serve as XEC partners with **1**, while most of them were efficiently converted to the desired enantioenriched products with excellent enantioselectivity. As for the alkyl halide scope, secondary as well as tertiary amides provided the corresponding products in moderate to good yields and enantioselectivities.

To conclude, Fu et al. have demonstrated a completely new strategy for performing enantioconvergent C*sp* ^3^−C*sp* ^3^ bond‐forming reactions empowered by photoexcited enzymes. While the scope was demonstrated for a wide range of coupling partners, it is expected that this concept will further leverage unprecedented chemo‐ and enantioselective *sp* ^*3*^‐*sp* ^*3*^ XEC. As this reactivity is unknown in small‐molecule catalysis, it demonstrates the power of accessing unnatural enzymatic reactions by exploring the interface between photochemistry and protein science. Moreover, this striking example further highlights that previous limitations in the reaction scope of enzymes are more and more overcome, thereby addressing long‐standing synthetic challenges.

## Conflict of interest

The authors declare no conflict of interest.

## References

[1] T. A. Scott , J. Piel , Nat. Chem. Rev. 2019, 3, 404–425. 10.1038/s41570-019-0107-1 PMC7104373 32232178

[2] B. A. Sandoval , T. K. Hyster , Curr. Opin. Chem. Biol. 2020, 55, 45–51. 31935627 10.1016/j.cbpa.2019.12.006 PMC7769163

[3] G. M. Whitesides , M. E. Wilson , J. Am. Chem. Soc. 1978, 100, 306–307.

[4] H. J. Davis , T. R. Ward , ACS Cent. Sci. 2019, 5, 1120–1136. 31404244 10.1021/acscentsci.9b00397 PMC6661864

[5] N. P. Dunham , F. H. Arnold , ACS Catal. 2020, 10, 12239–12255. 33282461 10.1021/acscatal.0c03606 PMC7710332

[6] S. B. J. Kan , R. D. Lewis , K. Chen , F. H. Arnold , Science 2016, 354, 1048–1051. 27885032 10.1126/science.aah6219 PMC5243118

[7] P. S. Coelho , E. M. Brustad , A. Kannan , F. H. Arnold , Science 2013, 339, 307–310. 23258409 10.1126/science.1231434

[8] C. C. Farwell , R. K. Zhang , J. A. McIntosh , T. K. Hyster , F. H. Arnold , ACS Cent. Sci. 2015, 1, 89–93. 26405689 10.1021/acscentsci.5b00056 PMC4571169

[9] R. Blomberg , H. Kries , D. M. Pinkas , P. R. E. Mittl , M. G. Grütter , H. K. Privett , S. L. Mayo , D. Hilvert , Nature 2013, 503, 418–421. 24132235 10.1038/nature12623

[10] W. Harrison , X. Huang , H. Zhao , Acc. Chem. Res. 2022, 55, 1087–1096. 35353478 10.1021/acs.accounts.1c00719

[11] M. J. Black , K. F. Biegasiewicz , A. J. Meichan , D. G. Oblinsky , B. Kudisch , G. D. Scholes , T. K. Hyster , Nat. Chem. 2020, 12, 71–75. 31792387 10.1038/s41557-019-0370-2 PMC6925616

[12] Z. C. Litman , Y. Wang , H. Zhao , J. F. Hartwig , Nature 2018, 560, 355–359. 30111790 10.1038/s41586-018-0413-7

[13] J. S. Trimble , R. Crawshaw , F. J. Hardy , C. W. Levy , M. J. B. Brown , D. E. Fuerst , D. J. Heyes , R. Obexer , A. P. Green , Nature 2022, 10.1038/s41586-022-05335-3. 36130727

[14] T. K. Hyster , Synlett 2020, 31, 248–254.

[15] H. Fu , J. Cao , T. Qiao , Y. Qi , S. J. Charnock , S. Garfinkle , T. K. Hyster , Nature 2022, 1–7, https://www.nature.com/articles/s41586-022-05167-1#citeas. 10.1038/s41586-022-05167-1 PMC10157439 35952713

